# Comprehensive multi-omics analysis of tryptophan metabolism-related gene expression signature to predict prognosis in gastric cancer

**DOI:** 10.3389/fphar.2023.1267186

**Published:** 2023-10-16

**Authors:** Peng Luo, Guojun Chen, Zhaoqi Shi, Jin Yang, Xianfa Wang, Junhai Pan, Linghua Zhu

**Affiliations:** Department of General Surgery, Sir Run Run Shaw Hospital, Zhejiang University School of Medicine, Hangzhou, China

**Keywords:** tryptophan metabolism, tumor microenvironment, immune cell infiltration, prognosis, gastric cancer

## Abstract

**Introduction:** The 5-year survival of gastric cancer (GC) patients with advanced stage remains poor. Some evidence has indicated that tryptophan metabolism may induce cancer progression through immunosuppressive responses and promote the malignancy of cancer cells. The role of tryptophan and its metabolism should be explored for an in-depth understanding of molecular mechanisms during GC development.

**Material and methods:** We utilized the Cancer Genome Atlas (TCGA) and Gene Expression Omnibus (GEO) dataset to screen tryptophan metabolism-associated genes via single sample gene set enrichment analysis (ssGSEA) and correlation analysis. Consensus clustering analysis was employed to construct different molecular subtypes. Most common differentially expressed genes (DEGs) were determined from the molecular subtypes. Univariate cox analysis as well as lasso were performed to establish a tryptophan metabolism-associated gene signature. Gene Set Enrichment Analysis (GSEA) was utilized to evaluate signaling pathways. ESTIMATE, ssGSEA, and TIDE were used for the evaluation of the gastric tumor microenvironment.

**Results:** Two tryptophan metabolism-associated gene molecular subtypes were constructed. Compared to the C2 subtype, the C1 subtype showed better prognosis with increased CD4 positive memory T cells as well as activated dendritic cells (DCs) infiltration and suppressed M2-phenotype macrophages inside the tumor microenvironment. The immune checkpoint was downregulated in the C1 subtype. A total of eight key genes, EFNA3, GPX3, RGS2, CXCR4, SGCE, ADH4, CST2, and GPC3, were screened for the establishment of a prognostic risk model.

**Conclusion:** This study concluded that the tryptophan metabolism-associated genes can be applied in GC prognostic prediction. The risk model established in the current study was highly accurate in GC survival prediction.

## Introduction

Gastric cancer (GC) has been recognized as a main cancer type that leads to cancer-associated mortality worldwide, with millions of new cases being diagnosed annually (Lambert et al., 2002; Brenner et al., 2009). Inflammation is typically related to GC development with both acute and chronic inflammatory cells, resulting in aggressive damage of gastric mucosa and ultimately transformation to cancer tissue (Demaria et al., 2010; Wang et al., 2014). In most cases, the 5-year survival of late-stage GC patients remains poor although current combination therapy of chemotherapy, radiation, and surgery has improved (Li et al., 2009; Song et al., 2017). Cancer immunotherapy has emerged recently as a promising and powerful cancer therapy that drives the patient’s own immune system against cancer (De Felice et al., 2015; Whiteside et al., 2016; Bruni et al., 2020). The combination therapy involving first-line Opdivo (nivolumab) immunotherapy and chemotherapy was approved in 2021 for advanced or metastatic GC patients (Twomey and Zhang, 2021; Yoneda et al., 2021). The mechanism of GC progression and its related tumor immune microenvironment should be analyzed to develop novel cancer immunotargets against GC.

As an essential amino acid, L-tryptophan serves as an indispensable material and regulates protein synthesis during cell proliferation (Conejos et al., 2021). Tryptophan and its metabolites play critical roles in various physiological processes (Hoseini et al., 2019; Conejos et al., 2021). Most free tryptophan is a biologically active substrate for the function of the kynurenine (Kyn) signaling pathway (KP) that produces several metabolites related to the immune response and neurotransmission (Platten et al., 2019; Tanaka et al., 2021). Many studies have focused on the imbalances in tryptophan metabolism by targeting the KP, especially ryptophan-2,3-dioxygenase (TDO), indoleamine-2,3,-dioxygenase 1 (IDO1), and IDO2 (Platten et al., 2019; Yao et al., 2021). It has been demonstrated that the tryptophan depletion by IDO1 and IDO2 was highly associated with cellular function and survival (Zhai et al., 2018; Souissi et al., 2022). However, phase III clinical trials of IDO inhibitors against cancers were disappointing, although they did show promising outcome in early-stage cancer immunotherapy (Günther et al., 2019; Chen et al., 2021). Some evidence has indicated that tryptophan metabolism may induce cancer development and progression through the inhibition of immune responses in the tumor site and promoting the malignancy of cancer cells (Platten et al., 2019). Although it is still unclear whether KP-related enzymes are essential for cancer progression, the role of tryptophan and its metabolism should be explored for in-depth understanding of molecular mechanism during GC development.

Thus, in this study, we used genes significantly associated with tryptophan metabolism pathway score to determine the molecular subtypes. Specifically, consensus clustering followed by subsequent comparison of clinical signatures, different signaling pathways, and immune-related properties among different subtypes will be performed. We then identified genes associated with the tryptophan metabolism phenotype by differential expression analysis and LASSO. We further predict GC patients’ outcome by constructing a risk model, which is also used for personalized treatment.

## Materials and methods

### Data sources, collection, and preprocessing

The data related to mutation, copy number variation, and RNA-Seq profile of TCGA-STAD via TCGA GDC API were downloaded first (http://cancergenome.nih.gov/). GSE66229 expression data from the GEO database were obtained (https://www.ncbi.nlm.nih.gov/geo/). Samples need to be processed as follows (Brenner et al., 2009): excision of samples of primary tumors (Lambert et al., 2002); removal of incomplete samples with clinical characteristic information to ensure that samples have complete clinical prognostic information and transcriptome expression data. TCGA-STAD was the training set and the GSE66229 dataset was the validation set. We excluded samples without survival time or status. Finally, a sum of 350 primary tumor samples together with 32 normal matches were screened. We kept the protein-encoding genes for TCGA RNA-seq data analysis. Meanwhile, all data were log2 transformed, and RNA expression data were normalized. For the GEO dataset, 300 GC samples were finally screened. Specifically, the annotation information for each chip platform was acquired and subsequently utilized to map probes with all the detected genes. Then, we removed the probes that matched more than one gene. When one gene can be matched by more than one probes, the mean value of the gene expression was calculated and set as the value for that specific gene.

The tryptophan metabolism-related gene information is derived from a specific pathway named “KEGG_TRYPTOPHAN_METABOLISM”, which can be found in the Molecular Signatures Database (MSigDB) (https://www.gsea-msigdb.org/).

### Molecular subtypes of tryptophan metabolism-associated genes

We constructed a consensus matrix and clustered the samples through consensus clustering (Wilkerson and Hayes, 2010). The transcriptional expressions of prognostic genes, which are involved in the tryptophan metabolism score, were subsequently evaluated to determine the molecular subtypes. We executed 500 bootstraps employing the “hc” algorithm and “pearson” as the metric distance. Each bootstrap process involved around 80% of the training set patients. The cluster number was set within a range of 2–10, and we determined the optimal classification via cumulative distribution function (CDF). Notably, the consistency of CDF was carefully evaluated when constructing various molecular subtypes for GC samples.

### Risk model

The distinctly expressed genes were identified among the molecular subtypes, and then the distinctly expressed genes associated with statistical significance for prognosis were sorted out (|Log2FC|>1 and FDR<0.05). The gene numbers were further condensed by Lasso regression, and the prognostic genes potentially contributing to the tryptophan metabolism phenotype were filtered. Then, the risk score for each patient was calculated by the formula:

RiskScore = Σβi×Expi, where Expi is the expression value of each prognostic gene that determines tryptophan metabolism phenotype. The Cox regression coefficient of corresponding prognostic gene is referred to as β. Based on the calculated numbers, samples were then distributed into two subgroups, that is, RiskScore-high and RiskScore-low groups, with the threshold set as “0". The commonly used Kaplan-Meier method was utilized to analyze patient survival, and the patients’ statistical significance was calculated via a log-rank test.

### GSEA

In different molecular subtypes, pathways of different biological processes were investigated by performing GSEA for signaling pathway analysis based on the candidate gene sets from the KEGG/hallmark (https://www.gsea-msigdb.org/gsea/index.jsp).

### Calculation of cell invasion abundance in tumor microenvironment

Relative abundance of 22 immune cells in tumor tissues and percentage of immune cells was determined using CIBERSORT algorithm (https://cibersort.stanford.edu/) and ESTIMATE software (Wilkerson and Hayes, 2010), respectively. A total of 28 immune cells were scored using ssGSEA function (Charoentong et al., 2017).

### Prediction of patients’ responsiveness to immunotherapy

The TIDE, as a widely used algorithm for immune checkpoint blockade (ICB) responsiveness prediction (Thorsson et al., 2018), was applied to verify the prediction of clinical responsiveness to ICB, which evaluated various cell types including tumor-related fibroblasts, which are responsible for excessive extracellular matrix deposition, immunosuppressive cells such as the M2 subtype of tumor-associated macrophages, and myeloid-derived suppressor cells that suppressed the T cell infiltration inside the tumor microenvironment, and two distinct mechanisms involved in escaping immune surveillance, including the score determining the dysfunctionality of tumor-infiltrating cytotoxic T lymphocytes (CTLs) and the score showing the rejection of CTLs by immunosuppressive factors.

## Results

### Genetic variation landscape of tryptophan metabolism-associated genes in GC

**FIGURE 1:**
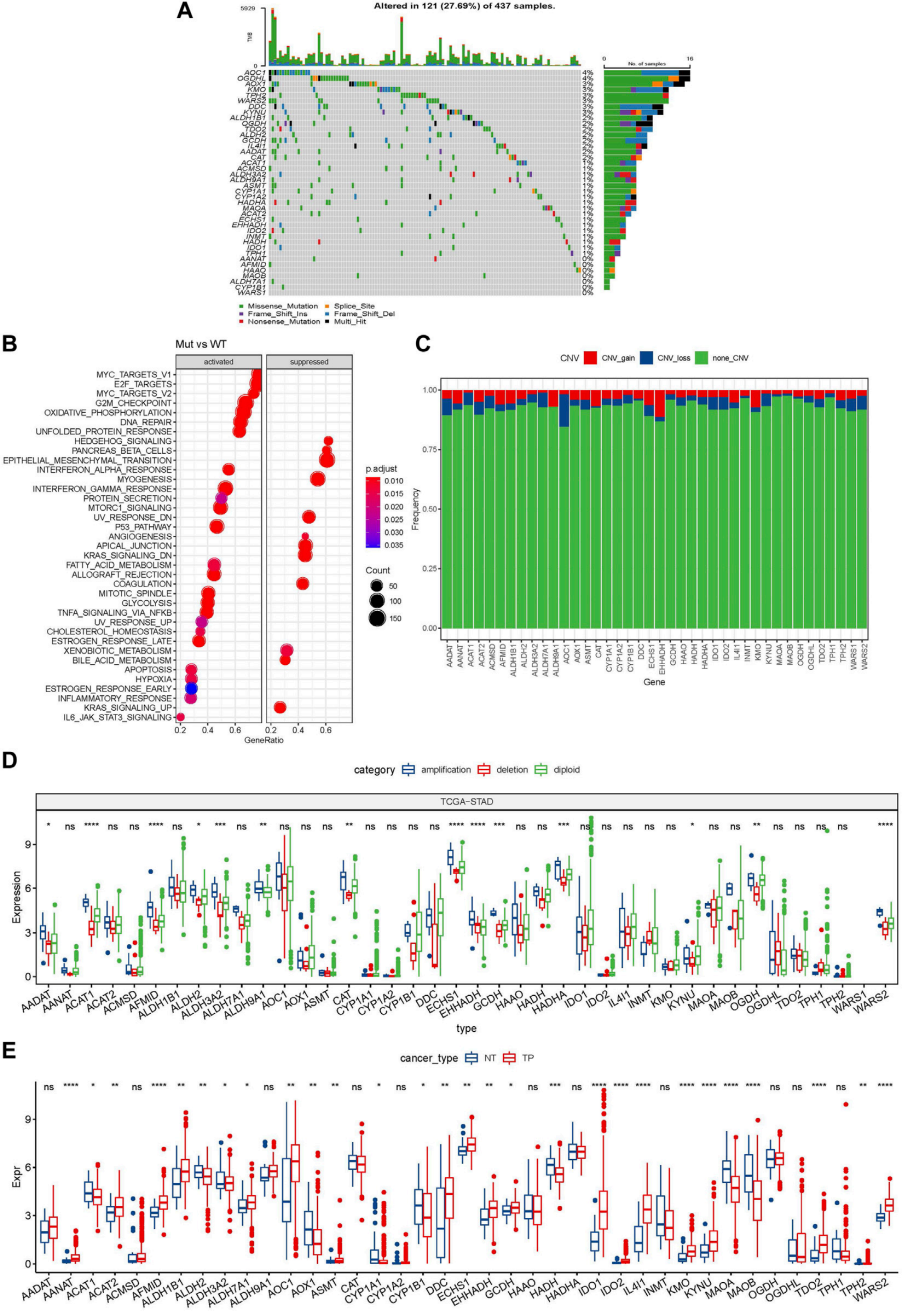
Genetic variation landscape of tryptophan metabolism-related genes in GC. **(A)** Mutational map of genes associated with tryptophan metabolism in primary tumor samples; **(B)** Primary tumor samples GSEA analysis between mutated and non-mutated groups; **(C)** CNVs of genes associated with tryptophan metabolism within primary tumor samples; **(D)** Gene expression between different types of copy number variation in primary tumor samples; **(E)** Differential expression of tryptophan metabolism-related genes between tumor and normal tissue samples. * means *p*-value less than 0.05; ** means *p*-value less than 0.01; *** means *p*-value less than 0.001, and **** means *p*-value less than 0.0001. ns means there is no significant difference between the two groups. The same statistical criteria apply to the following figures.

A total of 40 genes were involved in tryptophan metabolism. To determine the genetic alteration of tryptophan metabolism in GC, mutation frequency of cells was analyzed among the tryptophan metabolism-associated genes. Among the 437 tumor samples, 121 (27.69%) samples had tryptophan metabolism mutations. As shown in Figure 1A, AOX1 and OGDHL genes had the highest mutation frequencies, and no mutation was found in the WARS1 gene. To understand the effect of mutations on tryptophan metabolism-related genes, we analyzed the biological signaling pathways in wild-type (WT) and mutant (Mut) groups through GSEA enrichment analysis. It shows that tumor-associated pathways, including TNFA_SIGNALING_VIA_NFKB, P53_PATHWAY, and MYC_TARGET, were enriched in the mutant group (Sanchez-Vega et al., 2018) (Figure 1B). We then examined the somatic copy number variations of these tryptophan metabolism-associated genes in GC tumor samples and discovered a lower frequency of copy number variation (CNV) deletion or amplification (Figure 1C). In order to explore mRNA expression of CNV value in tumor tissue, the samples were distributed into different groups relying on CNV value, including increase and loss of CNV, as well as no obvious variation in CNV. Comparison of the expression of genes correlating with tryptophan metabolism between these groups showed that patients with CNV gain had a higher mRNA expression level compared to those with CNV loss (Figure 1D). To determine the expression of the tryptophan metabolism-associated genes between tumor tissue samples and adjacent normal tissues. As indicated in Figure 1E, most tryptophan metabolism genes were significantly differentially expressed, such as AANAT, AFMID, HADH, IDO1, IDO2, IL4I1, KMO, KYNU, MAOA, MAOB, TDO2, and WARS2.

### Molecular subtyping based on genes related to tryptophan metabolism

**FIGURE 2:**
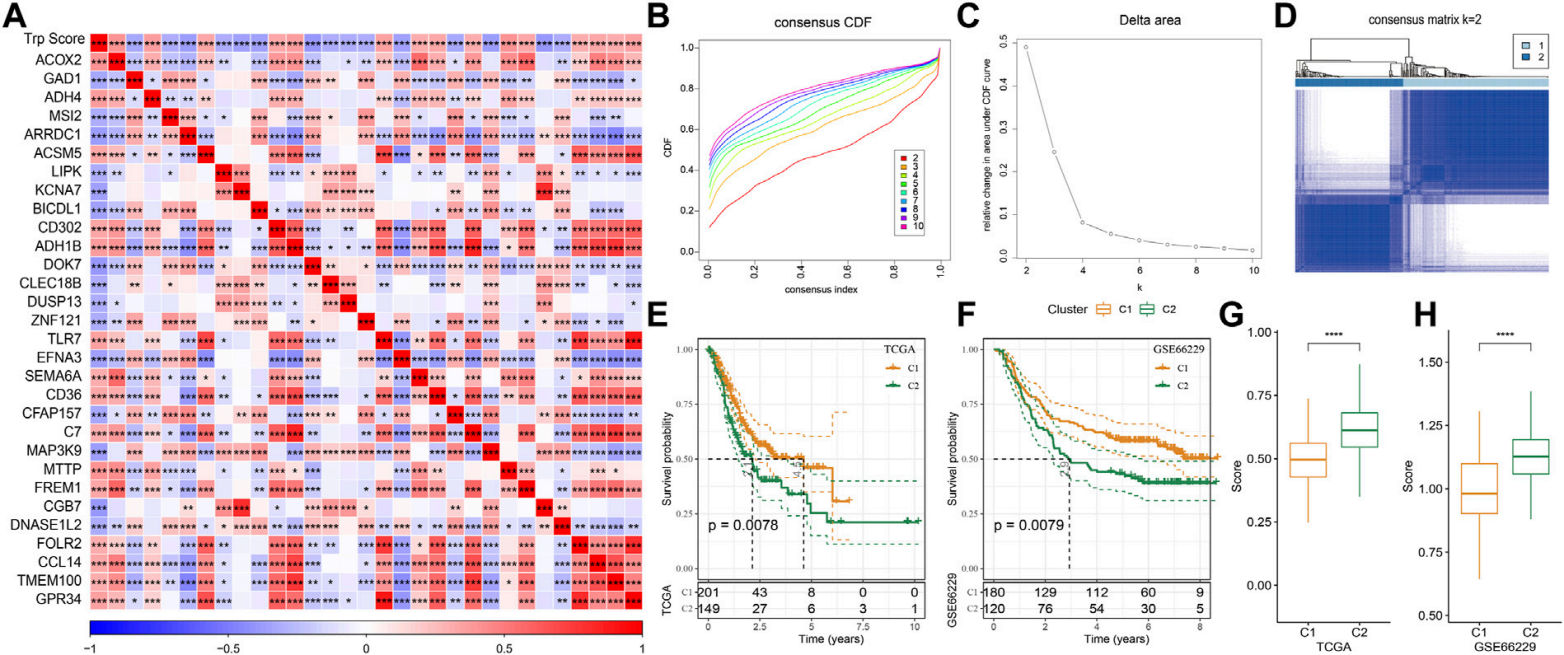
Molecular typing on genes that are associated with tryptophan metabolism. **(A)** The result of correlation analysis on genes that are significantly and prognostically associated with tryptophan metabolic pathway scores is summarized into a heatmap; **(B)** Cumulative distribution function curves for samples that are from TCGA cohorts; **(C)**. Curves for the delta area under the cumulative distribution function curves for samples that are from TCGA cohorts; **(D)**. The second sample clustering (k = 2) is displayed as a heatmap; **(E)**. The prognosis of two TCGA subtypes is displayed as a KM curve; **(F)** The prognosis of the two GSE66229 cohort subtypes is displayed as a KM curve; **(G,H)**: The statistical differences of tryptophan metabolism scores among different molecular subtypes in the TCGA cohort **(G)** and in the GSE66229 cohort **(H)** were analyzed by one-way ANOVA.

**FIGURE 3:**
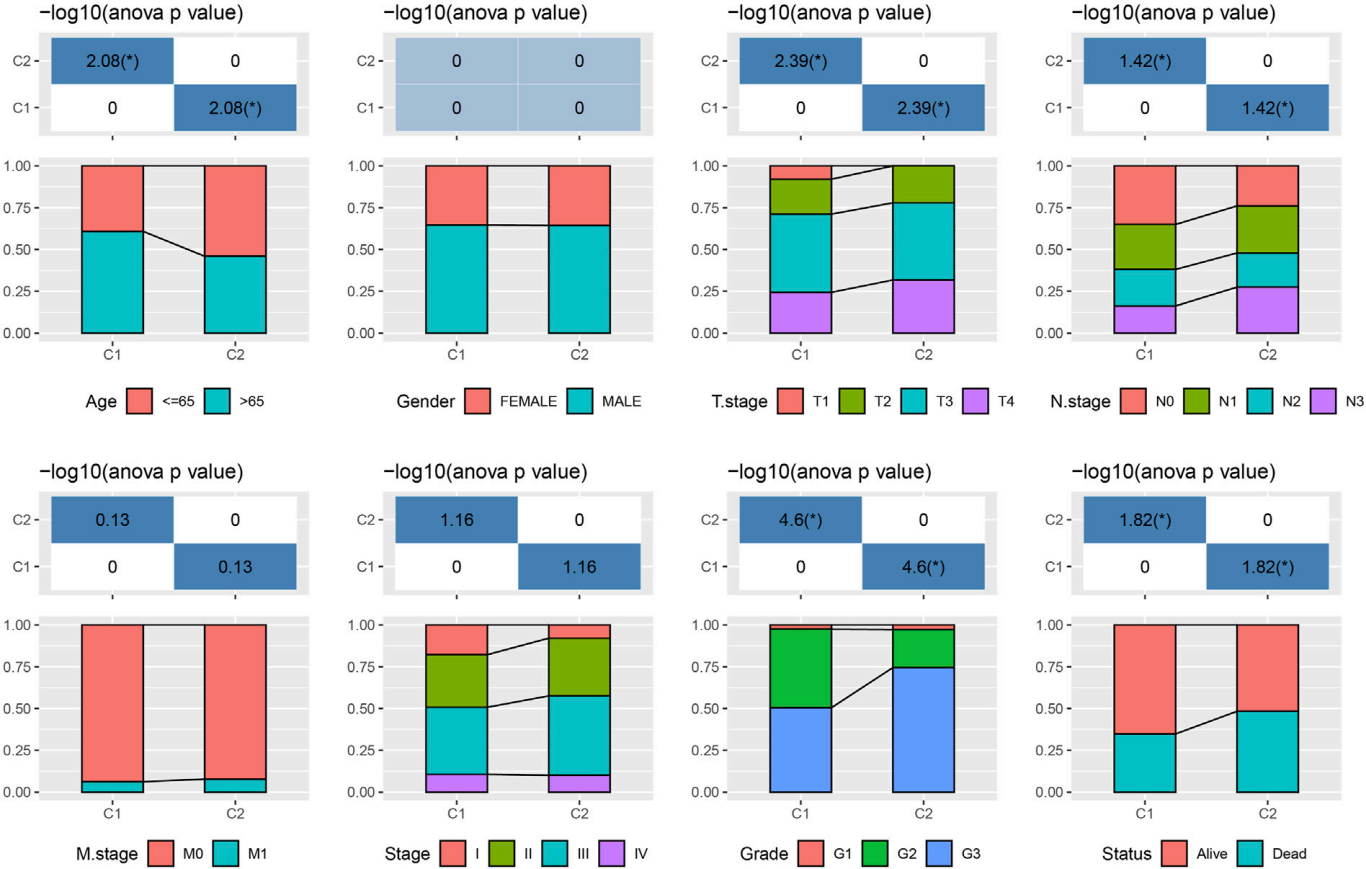
Clinical information distribution of molecular subtypes for the TCGA cohort.

The tryptophan metabolism score in the TCGA dataset was calculated by the ssGSEA, and then Pearson was used to estimate the relationship between the protein-encoding genes and the tryptophan metabolism score. A total, 30 prognostic genes associated with tryptophan metabolism score were screened. Figure 2A showed the correlation between the 30 genes and tryptophan metabolism scores. We classified patients based on the consensus clustering on 30 prognosis-correlated gene expression profiles and selected the optimal cluster number based on the CDF. With relatively stable clustering results shown in Figures 2B, C, we finally chose k = 2 to acquire two molecular subtypes (Figure 1D). We further performed the prognostic analysis of these two molecular subtypes. As shown in Figure 2E, we found that the overall survival of C1 was significantly better than that of C2. In addition, a sample of GC patients from the GSE66229 dataset showed similar results. This suggests that GC patients in C1 would have a better prognosis relative to the C2 subtype (Figure 2F). Meanwhile, we determined the tryptophan metabolism scores of each sample in the TCGA and GSE66229 datasets, which showed the score of C2 was higher compared to that of the C1 subtype with a good survival benefit (Figures 2G, H). Furthermore, differences in clinicopathological characteristics of TCGA molecular subtypes were analyzed. As indicated in Figure 3, we found significant differences between the two molecular subtypes in terms of age, disease stages, grade classification, and patient survival status.

### Various immune characteristics and responses to immunotherapy and chemotherapy between molecular subtypes.

**FIGURE 4:**
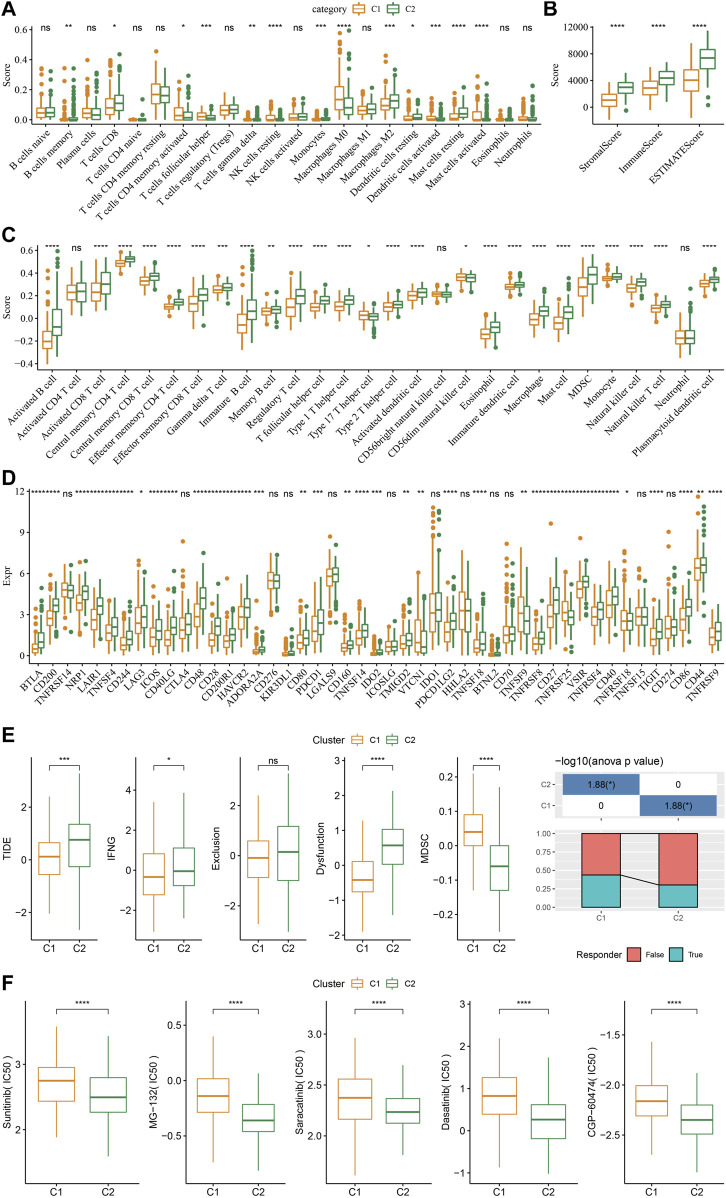
Differences in immune signatures between molecular subtypes treated with immunotherapy or chemotherapy. **(A)** The abundance of 22 immune cells shows differences among various molecular subtypes; **(B)** ESTIMATE immune infiltration differences among various molecular subtypes; **(C)** The scores for 28 immune cells vary among different molecular subtypes; **(D)** Differentially expressed immune checkpoints between different groups; **(E)** The results of TIDE analysis show significant differences comparing different TCGA cohort groups; **(F)** The estimated IC50 values for drugs in TCGA-STAD are displayed as box plots.

As shown in Figure 4A, some immune cell types were significantly different among the subtypes. Compared to the C2 subtype, activated dendritic cells (DCs) and CD4 positive memory T cells were significantly upregulated while the M2-phenotype macrophages were suppressed in the C1 subtype. Meanwhile, ESTIMATE was applied to evaluate the level of immune cells inside tumor tissues. Figure 4B showed that the ImmuneScore of the C2 subtype was significantly higher compared to that of other groups, indicating that C2 has a high level of immune cell infiltration. In addition, ssGSEA demonstrated significant differences in most immune cell scores between different subtypes (Figure 4C). We further analyzed the responsiveness to immunotherapy between different TCGA cohort molecular subtypes. As shown in Figure 4D, compared to C1 subtype, the gene expression of immune checkpoints such as IDO1, IDO2, and CD274 were increased dramatically in the C2 subtype.

We did this by employing the TIDE algorithm in order to assess the potential response of tumor samples to immune checkpoint inhibitors. Higher TIDE scores represent a higher likelihood of immune escape in response to immune checkpoint therapy (Jiang et al., 2018). As shown in Figure 4E, in the TCGA cohort, the C2 subtype showed a much higher TIDE score than the C1 subtype with best prognosis, indicating that the C2 subtype had greater potential of immune escape. In addition, analysis on the response of different molecular subtypes to conventional chemotherapeutic drugs showed that C2 patients were more sensitive to these drugs including sunitinib, MG-132, saracatinib, dasatinib, and CGP-60474 (Figure 4F).

### Mutational signatures and pathway analysis between molecular subtypes

**FIGURE 5:**
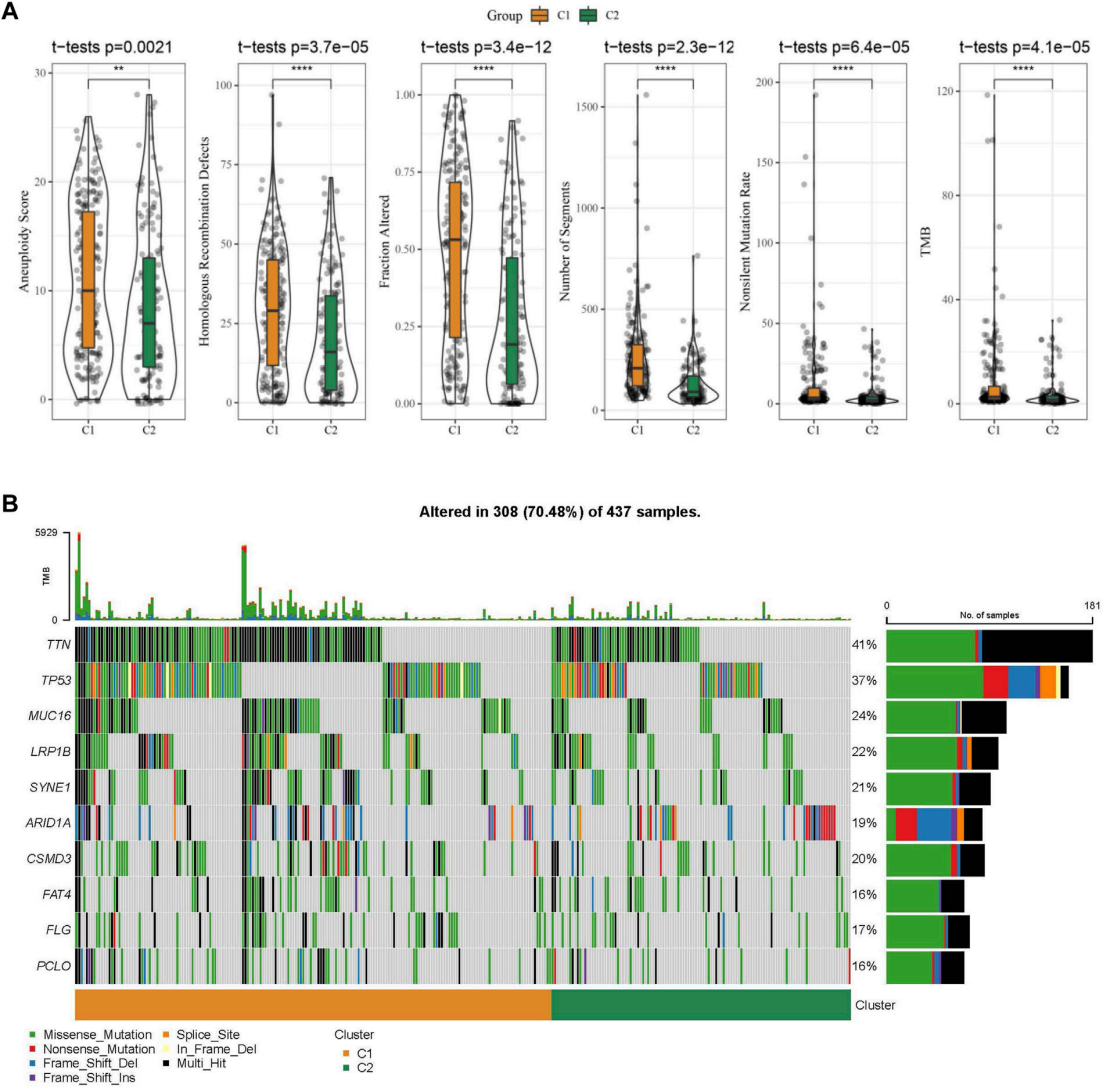
The molecular subtypes within the TCGA cohort show genomic variations. **(A)** Comparison of Homologous Recombination Defects, Aneuploidy Score, Fraction Altered, Number of Segments, Nonsilent Mutation Rate, and Tumor mutation burden in the TCGA cohort molecular subtypes; **(B)** Somatic mutations in the two molecular subtypes.

Further, differences in genomic alterations between two different molecular subtypes in the TCGA cohort were analyzed. Here, we obtained molecular information of TCGA-STAD collected from a pan-cancer study (Thorsson et al., 2018). The C1 subtype showed a higher aneuploidy score, fraction altered, homologous recombination defects, non-silent mutation rate, tumor mutation burden (TMB), and number of segments (Figure 5A). Gene mutation differences between different molecular subtypes were also studied. The top 10 genes were exhibited in Figure 5B, which showed significant differences of TTN, TP53, and MUC16 genes in mutation frequency between the two molecular subtypes. Similarly to our results, it has been reported that TTN, TP53 and MUC16 are the most significantly mutated driver genes in GC, which is closely related to the prognosis of cancer patients (Dong et al., 2022).

**FIGURE 6:**
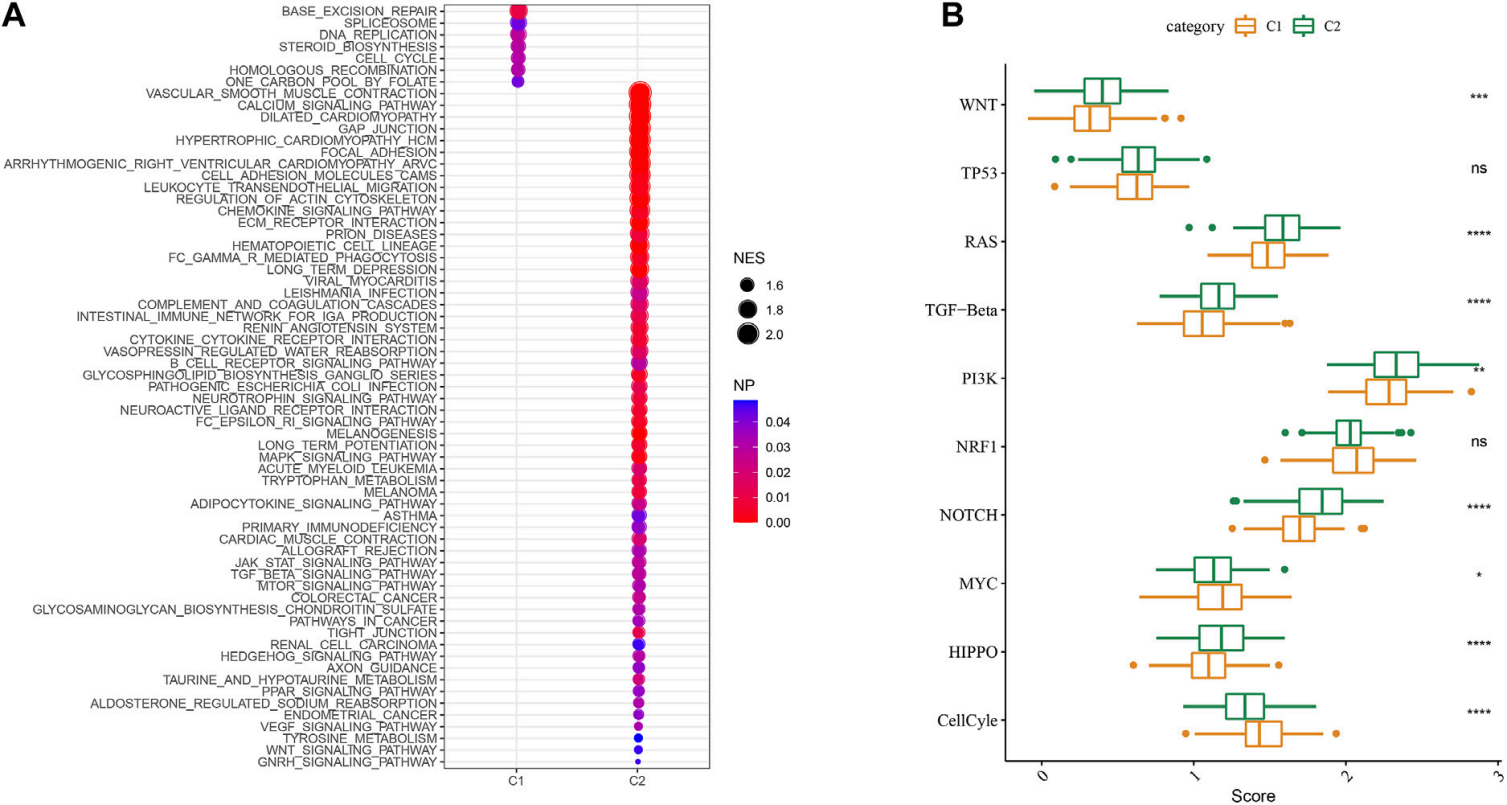
Signaling analysis between molecular subtypes. **(A)** The GSEA results of the TCGA cohort; **(B)** The scores of 10 signaling pathways associated with tumor abnormalities for various TCGA cohort molecular subtypes.

We then performed GSEA analysis which showed that DNA replication, spliceosome and base excision repair signaling pathways were highly enriched in the C1 subtype, while the C2 subtype had high enrichment of the phagocytosis, chemokine, and leukocyte transendothelial migration signaling pathways (Figure 6A). We also evaluated the 10 oncogenic pathways from the previous study (Sanchez-Vega et al., 2018). The differences showed statistical significance in the rest of the pathways except for the TP53 and NRF1 signaling pathways (Figure 6B). Compared to the C2 subtype, the oncogenic pathways, such as the Wnt, PI3K, and RAS pathways (Zhang et al., 2001; Zhang et al., 2019; BY et al., 2020), were significantly downregulated in the C1 subtype, which indicated an association between activated oncogenic pathways and tryptophan metabolism that may result in poor prognosis in C2.

### Identification of key genes for the tryptophan metabolism phenotype

**FIGURE 7:**
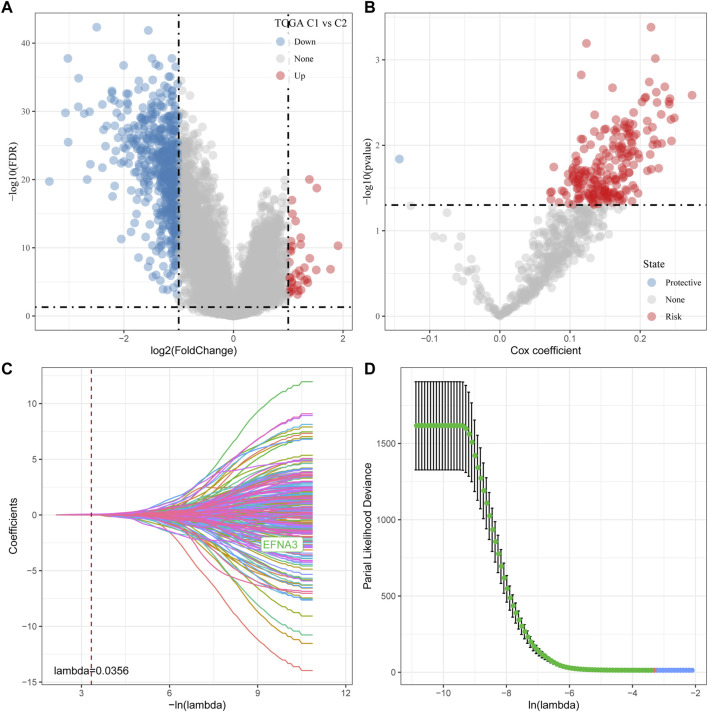
Determination of predominant genes contributing to phenotypes related to tryptophan metabolism. **(A)** Gene expression difference is displayed by a volcano plot; **(B)** A total of 218 potential candidates were determined among the differentially expressed genes; **(C)** Trajectory schemes were drawn for every independent variable associated with lambda; **(D)** Confidence interval under lambda.

As shown above, we identified two different molecular subtypes with distinct prognostic, immune, mutational, and pathway signatures. Next, we screened genes which are differentially expressed in C1 subtypes compared with C2 subtypes (|Log2FC| > 1; FDR <0.05), and a sum of 618 distinctly expressed genes were obtained as shown in the volcano plot of differential analysis (Figure 7A) in which 36 genes were significantly increased and 582 genes were decreased. We next utilized univariate cox analysis on the 618 differentially expressed genes and identified a total of 218 genes that showed the greater impact on prognosis, including 217 Risk genes and 1 Protective gene (Figure 7B). Subsequently, we compressed these 218 significant differentially expressed genes through lasso regression. As shown in Figures 7C, D, 10-fold cross-validation was used to construct the model and eight genes at lambda = 0.0356 were screened for further analysis: EFNA3, GPX3, RGS2, CXCR4, SGCE, ADH4, CST2, GPC3. The final 8-gene signature formula was as follows:

RiskScore=(0.024*EFNA3)+0.079*GPX3+0.102*RGS2+0.051*CXCR4+0.014*SGCE+0.076*ADH4+0.067*CST2+0.066*GPC3.

### Establishment and validation of clinical prognostic model

**FIGURE 8:**
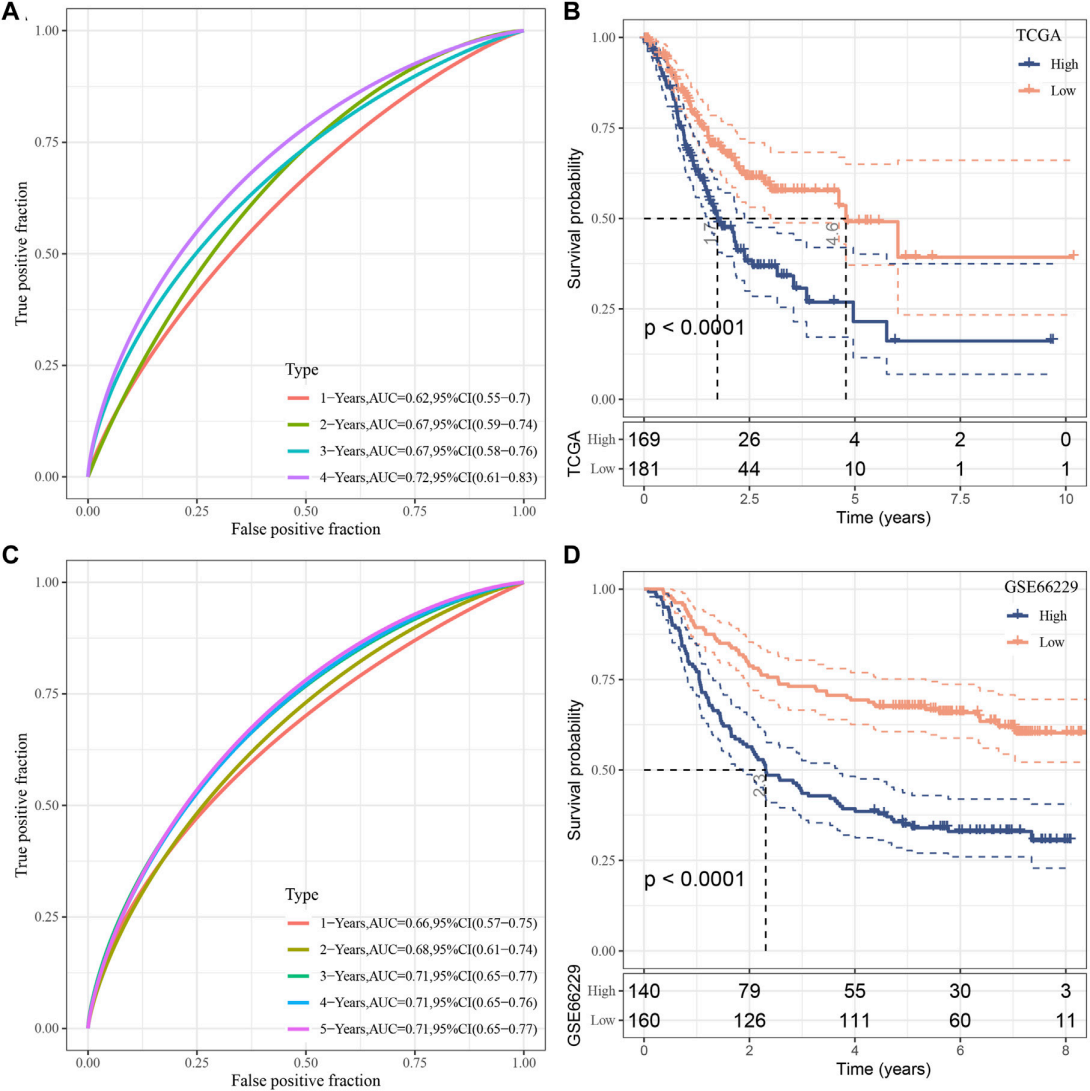
Establishment as well as validation of clinical prognostic model. **(A–B)** ROC curves and KM curves for eight genes derived from TCGA dataset; **(C–D)** ROC curves and KM curves for eight genes derived from GSE66229 dataset.

The risk score for each TCGA sample was separately calculated, followed by conducting a receiver operating characteristic (ROC) analysis on the prognostic classification of the RiskScore. The prediction classification efficiency from 1 year to 4 years was calculated to have an area under the time-dependent ROC curves (AUC) of 0.7, which validated the prediction capability of the model (Figure 8A). We then performed the zscore on the RiskScore. When the RiskScore was lower than zero, the samples were separated into the low-risk group, while those with a RiskScore higher than zero were in the high-risk group. As shown in Figure 8B, the low-risk group showed prolonged survival time, indicating the good performance of this prognostic model. Additionally, GSE66229 dataset was utilized to test the robustness and validate the risk model constructed by these eight genes. As shown in Figures 8C, D, similar results were observed, which indicated the excellent predictive ability of this model.

### RiskScore performance on clinicopathological features and different molecular subtypes

**FIGURE 9:**
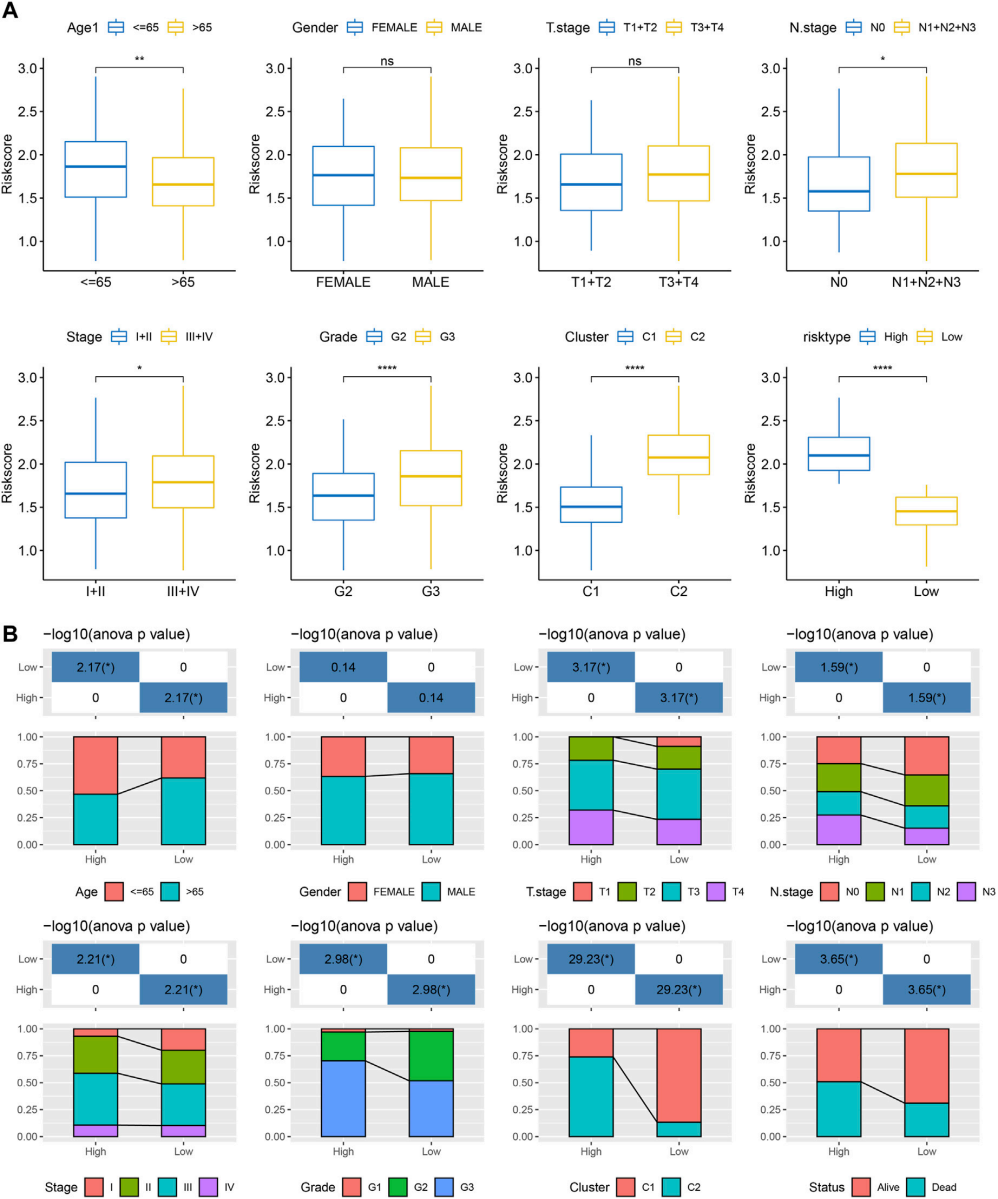
RiskScore performance on various clinicopathological features and various molecular subtypes. **(A)** Various clinicopathological groups derived from TCGA cohort are compared pairwise in the parameter of RiskScore; **(B)** Clinicopathological characteristics between RiskScore groups derived from TCGA cohort. G2 and G3 stages were selected for comparison. For M stage, there were 312 patients in M0 stage, and only 23 patients in M1 stage. Therefore, the difference in RiskScore between M stages was not compared in this study.

We analyzed the differences in RiskScore between Gender, Age, TNM grades, Stage clinical grades, and Grade grades in the TCGA-STAD dataset to explore the relationship between RiskScore and clinical characteristics of GC. With the increase in the clinical grade, the RiskScore also increased (Figure 9A). Moreover, we analyzed the difference in RiskScores among different molecular subtypes. The RiskScore of the C2 subtype with worse prognosis was obviously higher compared to that of the C1 molecular subtype with the best prognosis. Clinicopathological characteristics between the RiskScore groups in the TCGA-STAD cohort were analyzed, and the high-risk group was found to have a higher clinical grade (Figure 9B), which was consistent with previous results.

### Immune infiltration/pathway characteristics between RiskScore groups

**FIGURE 10:**
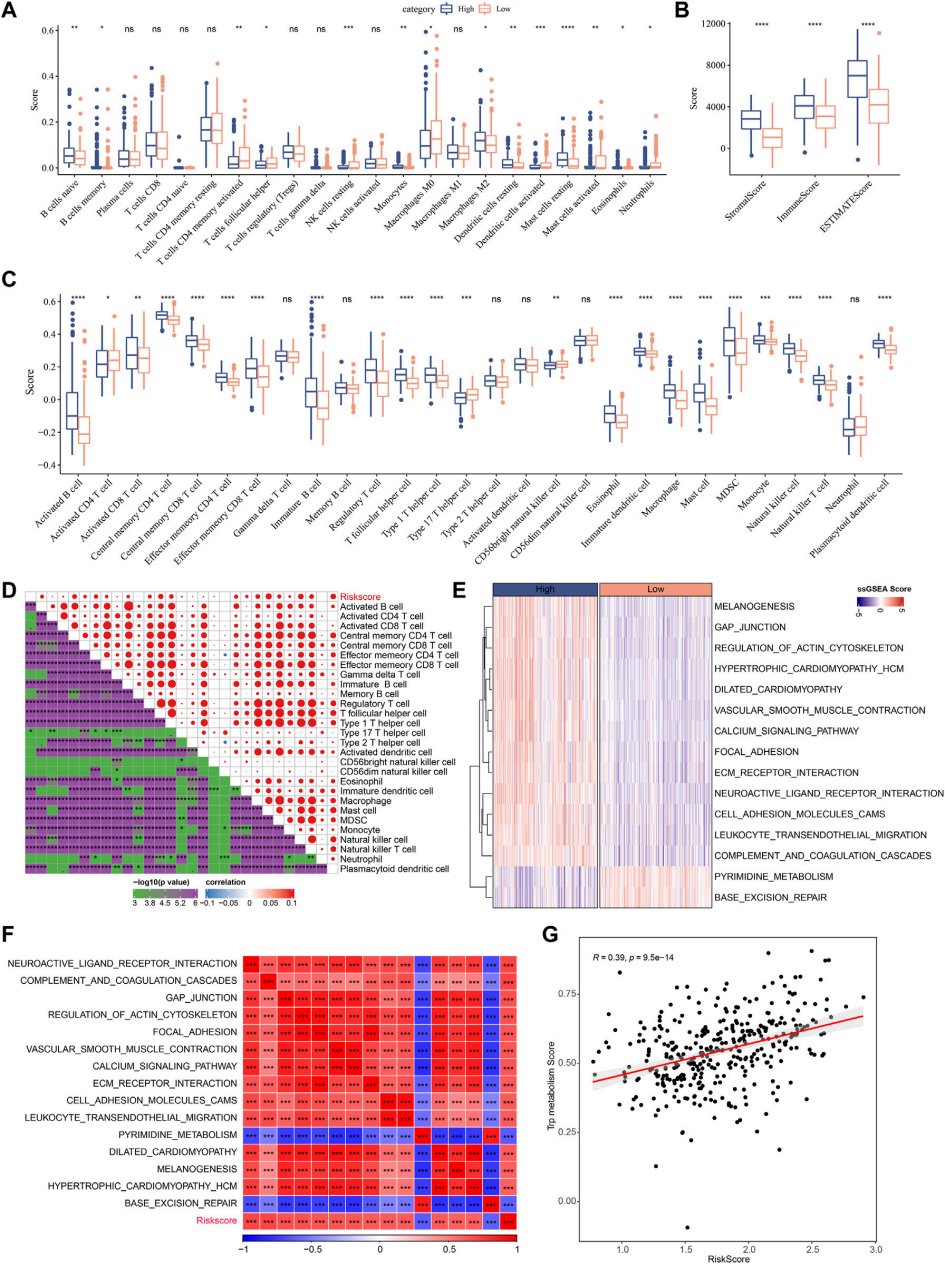
Immune infiltration/pathway characteristics between RiskScore groups. **(A)** The abundance of 22 immune cells in TCGA cohort between high and low risk groups is shown as boxplot; **(B)** Boxplot of differences in immune scores calculated by ESTIMATE software in TCGA cohort; **(C)** Boxplots of differences in 28 immune cell scores calculated by ssGSEA in TCGA cohort; **(D)** Correlation analysis between 28 immune cell scores and RiskScore in the TCGA cohort; **(E)** The enrichment scores for signaling pathways in high-risk and low-risk groups with a correlation factor larger than 0.6 are displayed as a heatmap; **(F)** Correlation relationship between GSEA pathways and RiskScore was performed and those with a correlation factor larger than 0.6 are displayed; **(G)** Correlation scatter plot between RiskScore and tryptophan scores.

Next, the enrichment of 22 immune cells in the high and low RiskScore groups was analyzed between RiskScore groups. As shown in Figure 10A, compared to the high-risk group, resting NK cells, activated CD4 T cells, and activated DCs were increased dramatically while M2 macrophages were substantially decreased in the low-risk group. ESTIMATE was also utilized to evaluate the level of immune cells in the tumor tissues. As shown in Figure 10B, the ImmuneScore in the high-risk group is much higher. Although it showed a higher level of immune cells in the high-risk group, the immunosuppressive cells such as MDSC and M2-phenotype macrophages also increased significantly, which might result in a poor prognosis. Further, we also used the ssGSEA function to analyze the scores of 28 types of immune cells, and most of the immune cell scores were significantly different between high- and low-risk groups (Figure 10C). The correlation between immune cells and RiskScore was further evaluated. As shown in Figure 10D, the RiskScore positively correlated with most immune cells, especially CD4 T cells, DCs, NK cells, and MDSCs, which could support the prediction of prognoses. This suggests that the infiltration of these immune cells increases as the risk score rises.

In Figures 10E–G, the most signaling pathways were positively correlated with the RiskScore of the samples and a significant positive correlation could be found between RiskScore and tryptophan metabolism ssGSEA scores, indicating that the activated tryptophan metabolism may induce poor prognosis.

### Differences in immunotherapy/chemotherapy between RiskScore groups

**FIGURE 11:**
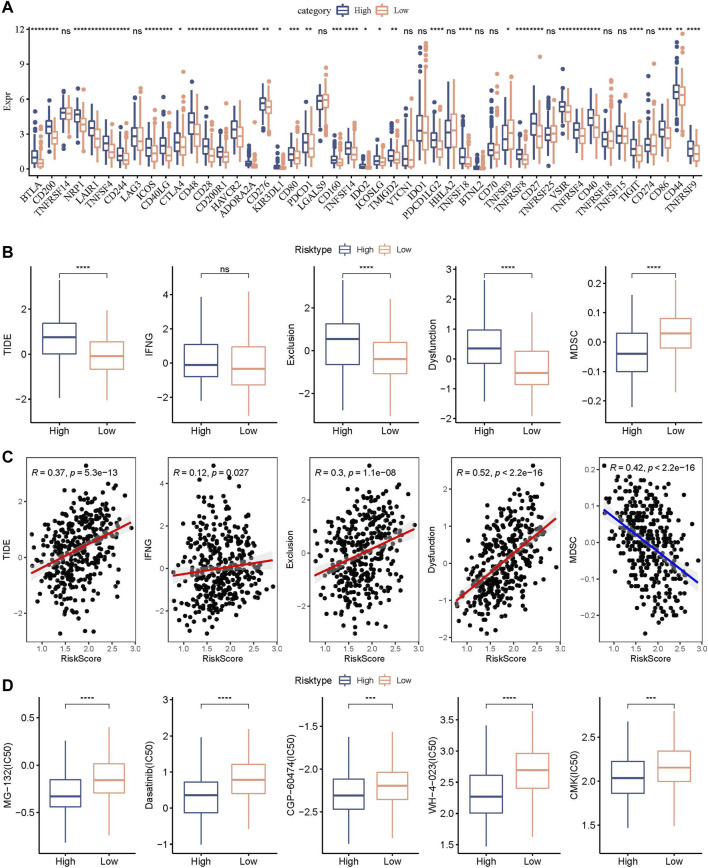
Genomic variations after immunotherapy and chemotherapy among RiskScore groups. **(A)** Differentially expressed immune checkpoint genes among different TCGA cohort groups; **(B)** The TIDE analysis results were compared between different TCGA cohort groups to show differences; **(C)** Within the TCGA cohort, a correlation relationship was performed between RiskScore and TIDE results; **(D)** The estimated IC50 values for drugs in TCGA-STAD were displayed as box plots.

We further explored the expression level of immune checkpoints between RiskScore groups. As shown in Figure 11A, some immune checkpoint genes, including NRP1, CD200, and CTLA4, were significantly downregulated in low-risk groups. We further analyzed the difference in immunotherapy among different RiskScore groups. TIDE software was applied to evaluate the immunotherapy response in the high and low RiskScore groups. We can find that in the TCGA cohort, the high-risk group showed a higher TIDE score (Figure 11B), indicating that the high-risk group showed greater potential of immune escape and less sensitive to immunotherapy. We further analyzed the relationship between RiskScore and TIDE score. Figure 11C showed a positive correlation between RiskScore and TIDE, IFNG, Exclusion, and Exclusion scores and a significant negative correlation with MDSC. In addition, we also analyzed the response of RiskScore groups to chemotherapeutic drugs in the TCGA cohort and found that the high RiskScore group was more sensitive to chemotherapeutic drugs including MG-132, dasatinib, CGP-60474, WH-4-023, and CMK (Figure 11D).

Combining RiskScore and clinicopathological features to optimize prognostic model and survival prediction.

**FIGURE 12:**
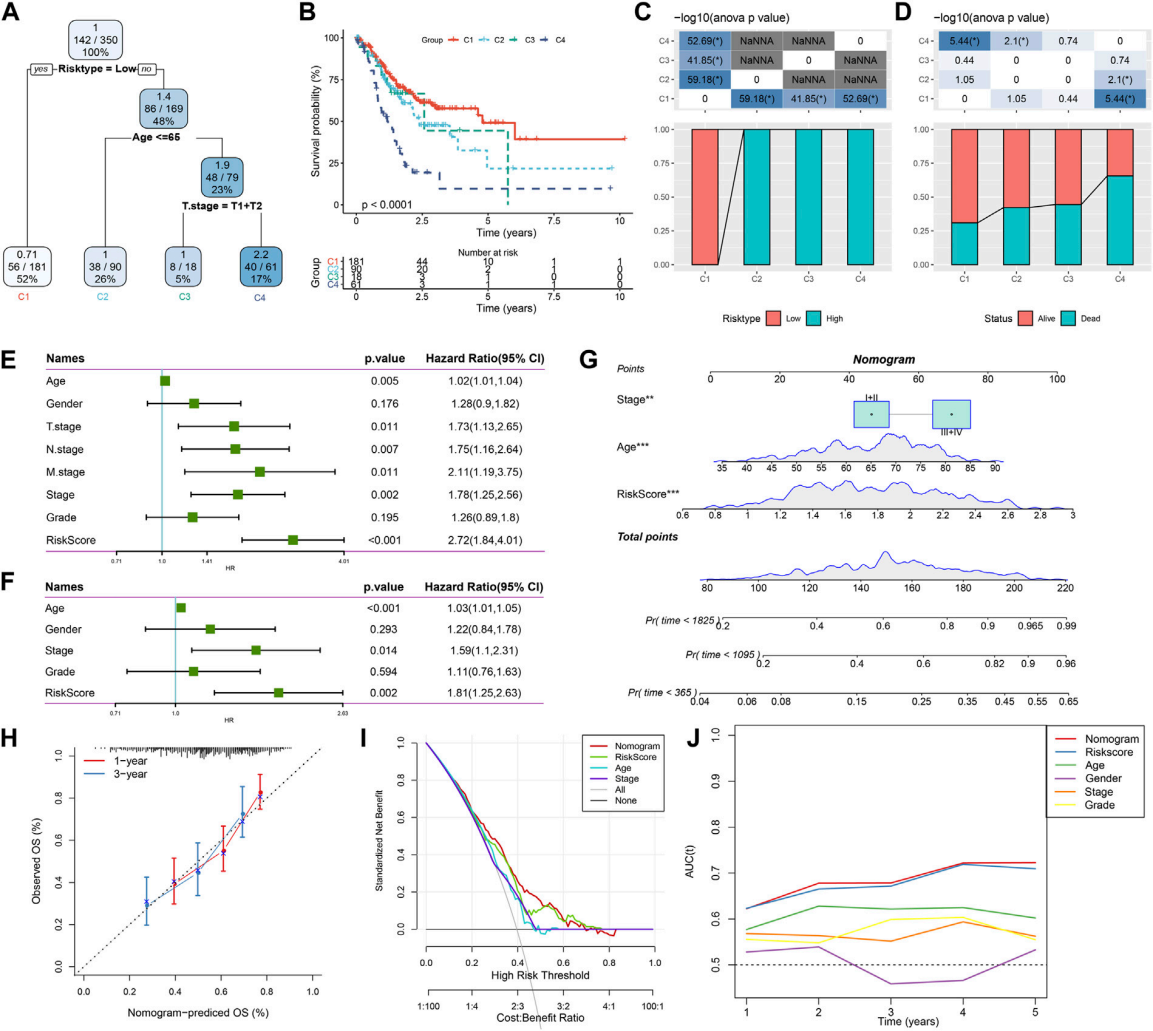
Combining RiskScore with clinicopathological features for the optimization of prognostic model and survival prediction. Patient samples with RiskScore, stage, and age were utilized to generate the survival decision tree; **(A,B)**: Overall survival among the different risk groups; **(C,D)**: Comparative analysis was performed between different groups; **(E)**: Univariate cox analysis of RiskScore and clinical features; **(F)**: Multivariate Cox analysis of RiskScore and clinical features; **(G)**: Nomogram model; **(H)**: Compared with other clinicopathological features, the nomogram showed the most powerful capacity for survival prediction; **(I)**: 1- and 3-year calibration curves of nomograms; **(J)**: Decision curves of nomograms.

Here, patient age, gender, TNM stage, Stage clinical grade, Grade, and RiskScore in the TCGA-STAD cohort were used to develop a decision tree. Only age, T stage, and RiskScore remained in the decision tree, where RiskScore was the most effective parameter (Figure 12A). Figure 12B showed a significant overall survival differences among the four risk subgroups. C2, C3, and C4 were all high-risk patients (Figure 12C). A significantly decreased survival benefit was found in the C2, C3, and C4 subgroups (Figure 12D). RiskScore, age, and Stage were significant prognostic factors, as confirmed by Univariate and multivariate Cox regression analysis on the clinical characteristics and RiskScore (Figures 12E, F). A nomogram combining the RiskScore with other clinicopathological characteristics was developed for the risk assessment and survival probability evaluation for patients. On survival rate prediction, the RiskScore showed the greatest impact. The accuracy of the prediction model was evaluated by the calibration curve (Figure 12G). As displayed in Figure 12H, the predicted calibration curve at 1 and 3 years(s) almost overlapped with the standard curve, indicating a strong prediction of the nomogram. Further decision curve analysis (DCA) demonstrated that the Riskscore and nomogram showed noticeably greater benefits than extreme curves. Additionally, both nomogram and RiskScore demonstrated the most powerful survival predictors compared to other clinicopathological features (Figures 12I, J).

## Discussion

Inflammation is typically associated with GC development and migration in damage of gastric mucosa (Demaria et al., 2010; Wang et al., 2014). The large number of immune cells, inflammatory cells and cytokines often present in the tumor microenvironment leads to a state of immunosuppression and chronic inflammation (Li et al., 2020). Although FDA approved combination therapy for the treatment of early stage and advanced GC patients recently, the 5-year survival of GC patients with advanced stage remains poor. We still need to investigate the mechanism of GC progression and its related tumor immune microenvironment for the development of novel cancer immunotargets against GC. Cancer is a typical metabolic disease. Nie et al. developed a prognostic model based on the metabolic profile of TCGA that can well predict the prognosis of STAD patients. This new metabolism-related feature can respond to the dysregulated STAD metabolic microenvironment (Nie et al., 2021). In addition, numerous studies have proved that the tryptophan metabolism could progress the GC development (Platten et al., 2019; Tanaka et al., 2021; Yao et al., 2021). Free tryptophan is a substrate for the kynurenine signaling pathway, which produces various metabolites related to the immune response. IDO1 and IDO2 were most popular rate-limiting enzymes to catabolize tryptophan, and many studies have focused on the blockade of IDO1 to active the antitumor immunity (Günther et al., 2019). However, phase III clinical studies of IDO inhibitors against cancers were unsatisfying (Chen et al., 2021). Although it is still unclear whether IDO enzymes are essential for cancer progression, tryptophan metabolism-related genes and signaling pathways were highly related to the GC immune microenvironment. Long et al. showed that tryptophan metabolism-related genes were significantly associated with immune infiltration of different cells in hepatocellular carcinoma (Long et al., 2023). Similarly, Zhang et al. showed that tryptophan metabolism-related genes play an important role in the immune microenvironment of gliomas. They constructed a tryptophan metabolism-related predictive model and found that higher tryptophan metabolism-related gene markers were significantly associated with immune cell infiltration (Zhang et al., 2022).

We therefore explored the molecular mechanism of tryptophan metabolism in GC by using RNA-seq data from human samples to establish the risk model used for predicting clinical results. Firstly, RNA-seq data derived from patients with GC were collected, and we analyzed the expression signatures and mutation profiles of tryptophan metabolism-associated genes. Then, two tryptophan metabolism-associated molecular subtypes were constructed to investigate the role of tryptophan metabolism in tumor immune microenvironment and further developed and verified the use of the model in a clinical setting.

It has been shown that Kynurenine, a catabolic metabolite of tryptophan, is able to bind to receptors for transcription factors, which in turn induces tumor cell invasion and immunosuppression of the tumor microenvironment (Xu et al., 2021). This suggests that amino acid metabolism plays a key role in the immunoregulatory mechanisms in tumor cells and the tumor microenvironment. In this study, we obtained a total of 40 genes that were highly related to tryptophan metabolism. Around 27.69% samples were found with tryptophan metabolism gene mutations, which was consistent with previous studies (Santhanam et al., 2016; Lu et al., 2020; Pirzadeh et al., 2022). This study classified two molecular subtypes and found that the C1 subtype showed better prognosis compared with the C2 subtype. But the immune cell infiltration and the ratio of some key immune activation cells especially naïve and memory CD8 T cells in the C1 molecular subtype were significantly more suppressed than those in C2 subtype. As demonstrated, CD8 T cell infiltration in the tumor microenvironment was critical in reviving antitumor immunity (Rahir and Moser, 2012; Chen et al., 2018). The suppressed immune cell infiltration in the C1 subtype might be induced by other immunosuppressive signals such as MDSC and the M2-phenotype macrophage, which were also important to form an immunosuppressive tumor microenvironment and inhibit the migration and penetration of immune cells into tumor tissues (Sica and Massarotti, 2017; Wang et al., 2021). We also analyzed the immune checkpoint gene expression in these two subtypes. Interestingly, more immune checkpoint genes were found to be suppressed in the C1 subtype compared with the C2 subtype, indicating that immune checkpoint blockade instead of immune cell infiltration resulted in a good clinical outcome for the C1 subtype. Additionally, the tumor-related signaling pathways such as the Nuclear Factor kappa B (NF-κB)/P53 pathway and MYC targets pathways were enriched in the mutant group. As reported, the NF-κB family was considered as a key regulator of immune responses and inflammation. Some literature has demonstrated that NF-κB/P53 signaling pathway activation was associated with human cancer development, progression, and metastatic potential (Tilborghs et al., 2017; Thorsson et al., 2018; Marei et al., 2021). Moreover, several studies have indicated that the transcription factor MYC served as a proto-oncogene in multiple cancers, which can result in transcriptional activation or repression of specific genes including those involved in tumor cell growth, proliferation, and survival (Zhang et al., 2010; Hu et al., 2018). The tumor-related signaling pathways enriched in the mutant group suggested that the mutation may result in functional changes and survival benefits for GC patients.

This study determined that eight significant genes (EFNA3, GPX3, RGS2, CXCR4, SGCE, ADH4, CST2, and GPC3) are correlated genes to construct the risk model. Among these genes, the chemokine receptor CXCR4 and its ligand CXCL12 were widely reported to be involved in cancer cell survival, proliferation, and migration (O'Boyle et al., 2013; Lee and Jo, 2012). Preliminary *in vivo* experiments suggested that CXCR4 might be essential in the development of a range of cancer malignancies (Conley-LaComb et al., 2013; Xue et al., 2017). The CXCR4/CXCL12 signaling could be considered as a therapeutic target in antitumor immunity and more in-depth exploration should be performed for the prediction of clinical outcomes. Moreover, some studies have demonstrated that glypican-3 (GPC3) was closely associated with tumor progression and acted as an oncogene in GC (Zhu et al., 2002; Ushiku et al., 2009), which was consistent with our findings. GPC3 might provide another potential therapeutic target for the treatment of GC.

We further established the risk model to predict the clinical outcome, which has been evaluated and verified with good performance and high survival prediction accuracy. The potential therapeutic targets among the tryptophan metabolism-related genes and signaling pathways could be applied in the clinical GC diagnosis and treatment. However, there are some limitations to note in this study. First, our study data were obtained from the TCGA and GEO databases, which were analyzed only by bioinformatics. In further studies, we should conduct relevant *in vivo* and *in vitro* validation experiments to verify the effect of risk modeling. In addition, the molecular mechanisms related to tryptophan metabolism in GC remain to be further verified.

## Conclusion

In this study, we screened and determined eight key genes that are related to the phenotype of tryptophan metabolism through differentially expressed gene analysis between molecular subtypes and constructed the risk model based on these key genes, which showed strong robustness and stable predictive performance with independence of clinicopathological characteristics. To optimize the risk model and prognostic prediction, we combined the RiskScore with clinicopathological features, which showed high accuracy and capability for survival prediction.

## Data Availability

The original contributions presented in the study are included in the article/supplementary materials, further inquiries can be directed to the corresponding author.

## Abbreviations

CNV, copy number variation; CDF, cumulative distribution function; FDR, false discovery rate; FC, fold change; GC, gastric cancer; GSEA, Gene Set Enrichment Analysis; ICB, immune checkpoint blockade; IDO, indoleamine-2,3,-dioxygenase; Kyn, kynurenine; KP, signaling pathway; MDSCs, myeloid-derived suppressor cells; ROC, receiver operating characteristic; WT, wild-type.
